# Cross-cultural emotion recognition and evaluation of Radboud faces database with an Indian sample

**DOI:** 10.1371/journal.pone.0203959

**Published:** 2018-10-01

**Authors:** Maruti Vijayshankar Mishra, Sonia Baloni Ray, Narayanan Srinivasan

**Affiliations:** Centre of Behavioural and Cognitive Sciences (CBCS), University of Allahabad, Allahabad, UP, India; Temple University, UNITED STATES

## Abstract

Emotional databases are important tools to study emotion recognition and their effects on various cognitive processes. Since, well-standardized large-scale emotional expression database is not available in India, we evaluated Radboud faces database (RaFD)—a freely available database of emotional facial expressions of adult Caucasian models, for Indian sample. Using the pictures from RaFD, we investigated the similarity and differences in self-reported ratings on emotion recognition accuracy as well as parameters of valence, clarity, genuineness, intensity and arousal of emotional expression, by following the same rating procedure as used for the validation of RaFD. We also systematically evaluated the universality hypothesis of emotion perception by analyzing differences in accuracy and ratings for different emotional parameters across Indian and Dutch participants. As the original Radboud database lacked arousal rating, we added this as a emotional parameter along with all other parameters. The results show that the overall accuracy of emotional expression recognition by Indian participants was high and very similar to the ratings from Dutch participants. However, there were significant cross-cultural differences in classification of emotion categories and their corresponding parameters. Indians rated certain expressions comparatively more genuine, higher in valence, and less intense in comparison to original Radboud ratings. The misclassifications/ confusion for specific emotional categories differed across the two cultures indicating subtle but significant differences between the cultures. In addition to understanding the nature of facial emotion recognition, this study also evaluates and enables the use of RaFD within Indian population.

## Introduction

In everyday social interactions, our decisions and actions are influenced by the facial expressions of the person with whom we communicate. Such emotional influences are studied using photographs of human models expressing distinct emotions. Unlike schematic faces, human facial stimuli offers the possibility of portraying a wider range of emotional expressions like disgust, fear, contempt, surprise, and sad with different levels of intensity. In addition, facial identity and gender can be manipulated providing greater, yet controlled degree of randomness and variability in the stimuli, which is an advantage not available with schematic faces.

The expression of emotions in humans is achieved via a complex combination of eyes, eyebrows, lips and facial muscles. Two standard guidelines had been proposed to categorize different facial expressions using a combination of these facial features: Izard’s [1] maximally discriminative facial movement coding system (MAX) and Ekman and Friesen’s [2] Facial Action Coding System (FACS). FACS is currently the most widely used method to portray basic facial expressions using facial muscle as action units, namely: happy, sad, surprise, angry, fear, disgust, contempt, and neutral. Using these guidelines various face databases have been developed; for example the NimStim database [3], Karolinska faces database [4], and Radboud faces database [5]. These databases consist of colour or gray scale pictures of people with different age groups, gender, and races (Asian, African, and Caucasian) portraying different expressions. These face databases are essential in investigating the fundamental questions about how emotions are perceived, recognized as well as to establish reciprocal interactions between affective and cognitive processes.

Face databases can aid in understanding emotion recognition across cultures. However, in order to use them, the same database needs to be evaluated within a particular culture. The evaluation should account for three important aspects: a) comparison of emotion recognition scores across the cultures (Is emotion recognition Universal?) b) given that each model would differ in the portrayal of emotional expression, how much ever trained, the models not recognized correctly should be eliminated from further studies within that culture, and c) when emotional stimuli are used across cultures, they need to be matched/ standardized on various parameters associated with emotional expressions like valence, intensity, genuineness, clarity and arousal. This is important since, many of these parameters lead to confound in controlled experimental designs. Validation of faces and expressions across cultures also helps in reducing the ambiguity associated with the images available in a particular database.

One of the central arguments in emotion literature, more specifically facial emotion expression, has been about the universality of emotion identification and recognition [6–8]. Of importance are the cross cultural studies that are a major contributing factor, either for or against universality. This issue, of whether emotional expressions are culture-specific or universal, has been debated for a long time [9–14]. Seminal research studies showed that basic emotions could be accurately recognized above chance across cultures [12,15]. However, it has been argued that most cross-cultural studies are confounded with cross-cultural contact, education, language, and familiarity It has also been reported that variations [16]. Variations in ethnicity, national and regional backgrounds, race, in-group versus out-group relations, facial display rules within a culture and social attitudes can influence emotion recognition across cultures [17–22].

To the best of our knowledge, very few studies have investigated cross-cultural emotion recognition with an Indian population. Elfenbein et al. [18] studied Indian, American and Japanese participants with photographs of facial expressions from the three cultural groups. While the photographs were generated from Indian and Japanese samples by asking models to display an expression by imagining an emotional scene (not following the FACS system), American posers followed the FACS manual for displaying prototypical emotional expressions. They reported that the trend of errors in emotion recognition were similar across the three cultures, partially supporting universality hypothesis but, also highlighted emotion specific cultural differences.

Cross-cultural emotional differences, especially in relation to display rules have also been studied in the context of either individualism or collectivism. In a broader sense, an individualistic culture endorses independence of an individual in a society, while a collectivistic culture supports group interactions and facilitates interdependence amongst its members [23,24]. These fundamental differences between the two types of cultures contribute to differences in general psycho-social attitudes [25–28] as well may contribute to differences in perception or ratings of emotional expressions as a function of whether they belong to in-group (their own region) or out-group members of a culture (other than their own region) [24,29]. For example, people in individualistic cultures (Americans) are more comfortable in displaying negative expressions than those from collectivistic cultures (Costa-Rica) [23,24]. A culture is not solely individualistic or collectivistic; nonetheless, Asians in general are considered more collectivistic than Western cultures in certain aspects [26]. India is not a purely collectivistic culture but rather shows features of both collectivism and individualism [24,25,27,28]. With Indian participants there are no measures of out-group facial emotion rating evaluated in the context of Individualism or collectivism. Given this background, we also wanted to check if rating and agreement rate data from Indian ratings for Radboud emotional faces (out-group) can be understood within the context of Individualism or collectivism.

Considering the above arguments, the motivation for this study was threefold. First, we aimed to test the universality of emotion recognition hypothesis in an Indian sample population from Allahabad with a full-fledged emotional database from another culture. At a broader level, we expected that there would be differences in subtle measures of emotion recognition like intensity, clarity etc. especially given that the faces belong to out-group members [16,30]. Second, we wanted to evaluate if differences in emotion recognition between Indian (out group) and Dutch (in group), follows those already reported for Individualistic or collectivistic cultures [21,22,24,29,31]. As Indians are reported to be a relatively more collectivistic culture than the Dutch [24], it could be expected that they would differ in agreement ratings for negative emotions in comparison to positive emotions, for out-group members. Third, we also aimed to validate the database for studies on emotions across cultures. To achieve this we selected the freely available Radboud Faces Database (RaFD) [5]. It offers ready to use colour pictures of Caucasian face stimuli of adults and children in three gaze directions and eight expressions: neutral, happy, angry, disgust, contempt, fear, surprise, and sad. All images were according to FACS guidelines and have been evaluated by taking ratings on parameters namely: valence, intensity, clarity, genuineness and correct identification of the expression [5]. For this study we selected only the adult facial expressions with frontal view and straight gaze direction.

Current research methodology is similar to that used by the developers of RaFD [5], in order to compare emotion rating and recognition differences between the two cultures (Indians and Dutch). In addition to the emotion categories and parameters originally used for RaFD, we also rated the database on ‘Arousal’ parameter, which is not available for RaFD. Emotions can be understood in terms of two parameters, namely valence and arousal [32,33], where valence could be positive or negative (pleasant/ unpleasant) and arousal represents the intensity of emotion felt by the participant (calm/ intense). Multiple studies suggest that emotion-cognition interactions are highly influenced by the arousal value of emotions than valence [34,35]. Given that arousal of an expression plays a significant role in emotion processing [36–38], having arousal ratings for this database would facilitate various cross cultural experimental studies in controlling for arousal values.

## Method

### Participants

Forty naïve observers (age range: 18–35 years, 25 females) with normal or corrected-to-normal vision provided informed written consent and participated in the experiment. All experimental protocols were approved by the Institutional Ethics Review Board of University of Allahabad.

### Apparatus

The stimuli were presented using E-Prime 2.0 Professional software [39] on a Samsung PC with windows (1024 x 768, 85 Hz) and the data was analyzed in Matlab [40] and R software [41].

### Stimuli

Only the front-faced straight gaze adult models from RaFD were used in this experiment. We used only seven expressions, namely; happy, angry, sad, surprise, disgust, neutral, and fear. We did not include ‘contempt’ expression, as it was the least accurately rated expression in the Radboud ratings. Moreover, the low accuracy rates for ‘contempt’ expression have been attributed to variations in facial features representing contempt expression across different cultures and regions [42,43].

A total of 39 models (19 females) each depicting the seven above mentioned expressions (273 images) were divided into two experimental sets (Set-1 & 2) of 19 and 20 models respectively (otherwise the duration of the experiment exceeded beyond two hours and technically not feasible to run on single participant). Set-1 had 133 and Set-2 had 140 images and the pictures were rated by two different groups of participants. The assignment of models into two groups was random. All expressions from each model were presented within one set only. Twenty participants rated each set. Each image was rated only once by a participant giving us twenty unique ratings for each image.

### Procedure

**Fig 1:**
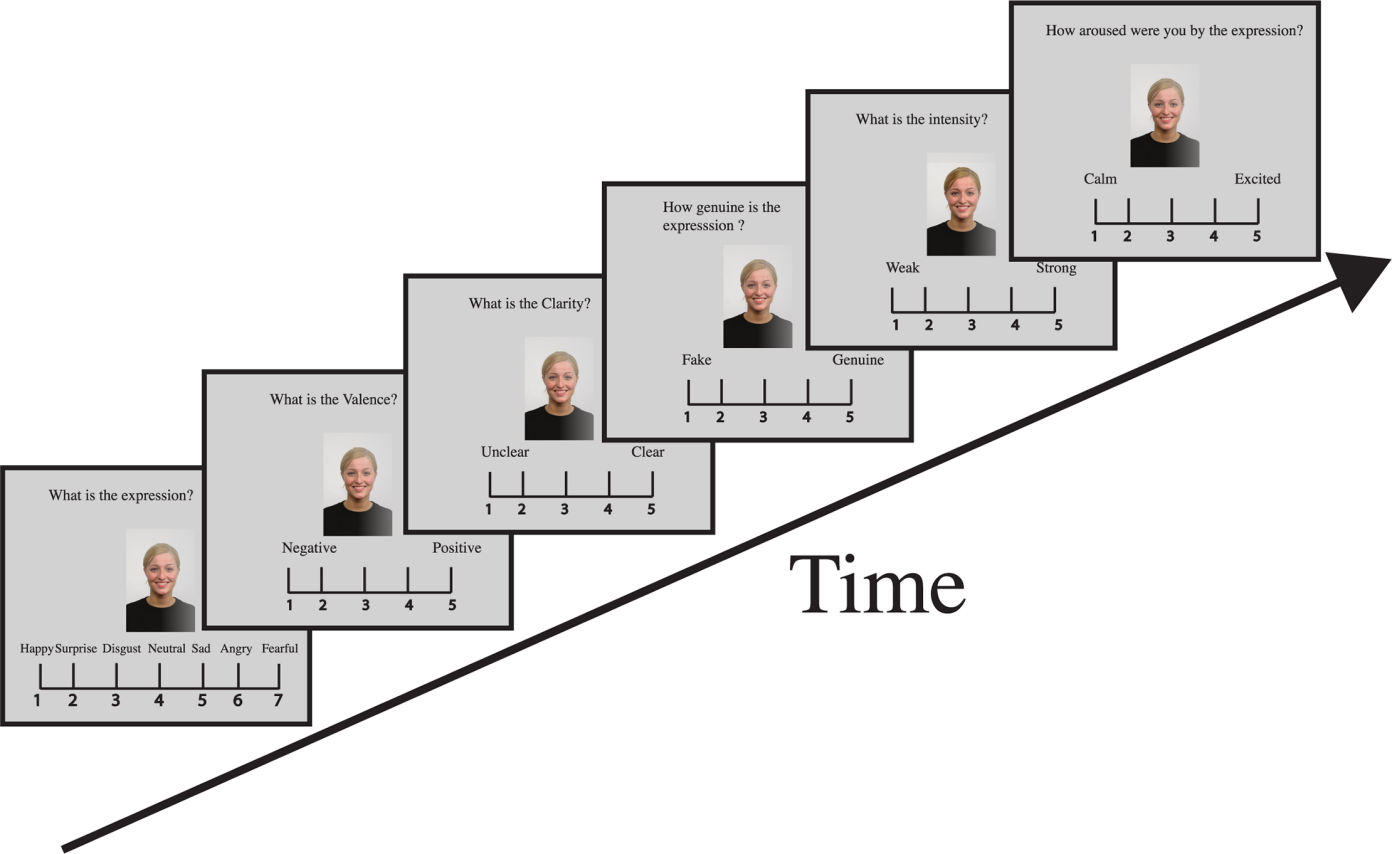
Single trial sequence for emotion rating. An Image was presented at the center of the monitor with the rating scale at the bottom and corresponding question at the top of the image. Participants were instructed to classify the emotion portrayed by the image followed by rating the same image for the five parameters namely valence, clarity, genuineness, intensity and arousal on a five point Likert-type scale.

Each experimental set had two rating blocks presented sequentially across all the participants, namely attractiveness rating block and emotion rating block. Each trial began with the presentation of an image at the center of the screen, the task question above the image and the rating scale below the image (Fig 1). The images were present on the screen until the participant rated it. The participants entered the responses using a keyboard. For each model, all emotional expressions were presented sequentially then followed by next model and all its expressions. Model image order was randomized for both blocks.

Participants rated attractiveness on a 5-point Likert like scale (1- unattractive, 5 –attractive). Only neutral expression of each model was used in this task so that the participants become familiar with the images for a given set. Same models were used in the emotion-rating task. The first question was based on emotion categorization task, where the participants were instructed to report the intended expression of the image on a 7-point nominal scale (1- happy; 2- surprise; 3- disgust; 4- neutral; 5- sad; 6- angry; 7- fearful). The participants were instructed to choose the label that best described the expression. This emotion categorization task was different from the original [5] task in two ways. First, we did not include the contempt expression in the task, for reasons mentioned above. Second, for the emotion categorization task we did not have ‘others’ option as used in their study [5]. Most of the 'others' responses among Dutch raters in the original article [5] were for contempt expression and, since we dropped the ‘contempt’ expression, we also dropped the ‘others’ option as well [44]. Apart from these two differences all other rating scales were similar to original task [5]. After emotion categorization task, participants rated the valence of the expression (negative to positive), clarity of the expression (unclear to clear), genuineness (false to genuine), intensity (weak to strong), and arousal (calm to excited) on a 5-point Likert type scale (1 to 5), one after the other sequentially for the same model. As mentioned previously, one of our objectives was to test the universality hypothesis of emotion recognition, and to achieve that we requested and obtained the original classification and rating data of Dutch participants [5] from the authors.

## Results

### Attractiveness rating

**Fig 2:**
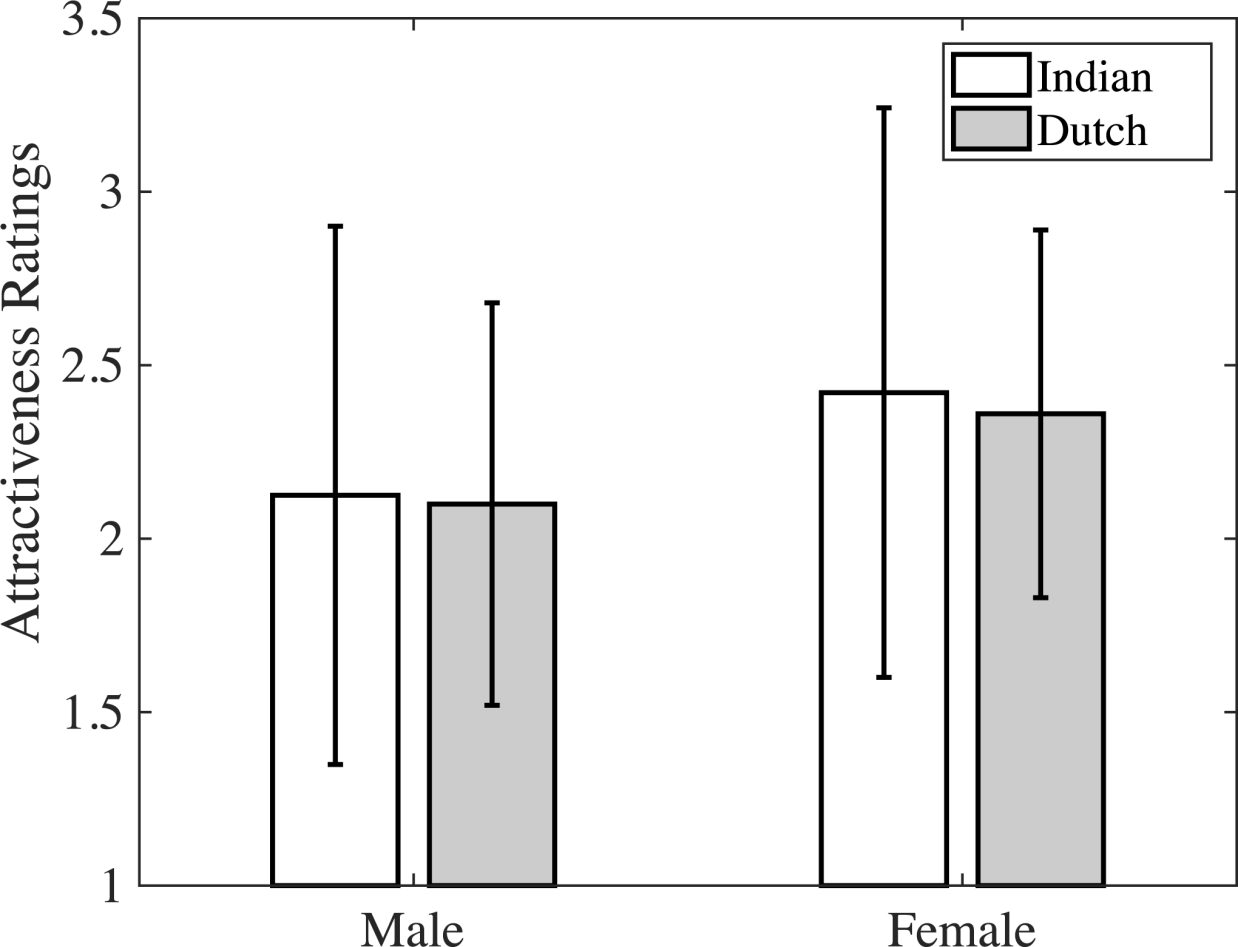
Attractiveness histogram. Bar plot comparing the mean attractiveness ratings for male and female models of Radboud database from Indian (white bar) and Dutch raters (grey bar). Error bars represents standard error of mean.

On a scale of 1–5, the mean attractiveness ratings for male and female adult models were not significantly different, *t*(37) = - 1.158, *p =* .254, CI = [-0.81 0.22]. Since we did not have individual values for attractiveness ratings from the Dutch participants, we were not able to do a statistical analysis comparing the attractiveness ratings for the two populations. But the mean ratings (mean ± standard deviation) of Indian and Dutch samples [5] for male (Indian = 2.13 ± 0.77, Dutch = 2.10 ± 0.58) and female (Indian = 2.42 ± 0.82, Dutch = 2.36 ± 0.53) adult models were similar across the two cultures (Fig 2).

### Expression agreement analysis

**Fig 3:**
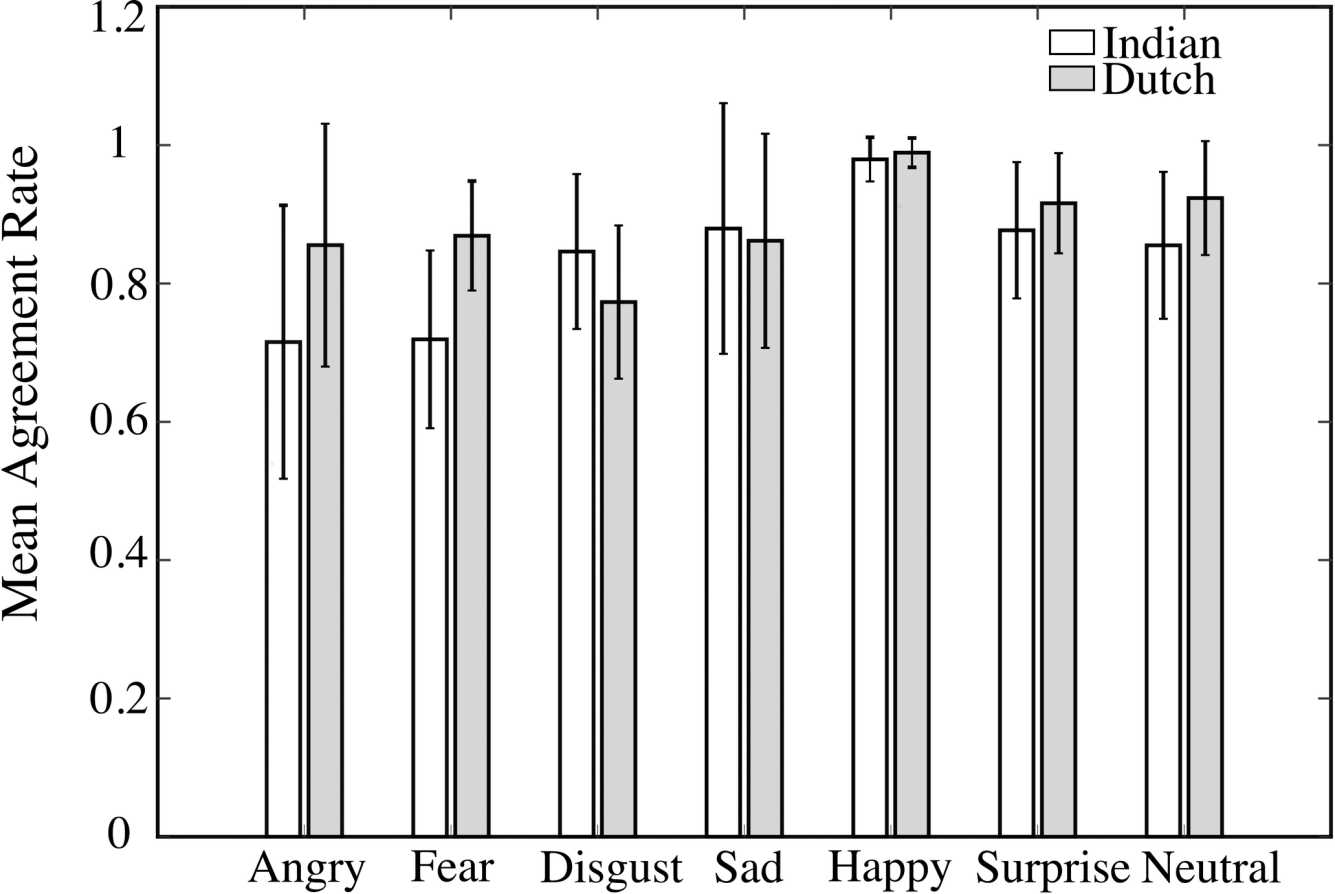
Agreement rate histogram. Bar plot comparing mean agreement rates for the seven expressions between Indian (White bars) and Dutch (Grey bars) participants. Error bars represent standard error of mean.

We evaluated the agreement rates, that is, the percentage of instances an emotion was correctly categorized as the intended expression (Fig 3). Overall (mean ± standard deviation) agreement rates across all emotion categories were 83.9% ± 15.7% (Median = 85%). Agreement rates for individual pictures by Indian and Dutch raters are provided in the supporting information (S1 Table). A one variable repeated measure (RM) ANOVA on arcsine-transformed agreement rates for the seven expressions was performed. The Mauchly’s test showed significant deviation of sphericity, W(6) = 0.26; *p* < .001, for expression (*ε*_Expression_ = 0.68), so Greenhouse-Geisser corrected values were used. The analysis showed significant effect of expression, *F*(4.08, 155.04) = 25.08; *p <* .0001, *η*_*p*_^*2*^ = 0.39. Post-hoc Tukey Kramer’s analysis showed that the agreement rates were significantly higher (all *p* < .001, all *Cohen’s d* = 1.17 ≤ d ≤ 1.93) for happy expression (*M* = 97.9%, *SD* = 3.2%) compared to all other expressions. The agreement rates for neutral, sad, surprise and disgust were not significantly different from each other (Fig 3). The agreement rates for angry (*M* = 71.5%, *SD* = 9.8%) and fear (*M* = 71.9%, *SD* = 12.8%) were the lowest and significantly differed from all other expressions (all *p <* 0.01; all *d* = ~ 1.9). In contrast, for the Dutch ratings, lowest agreement was observed for contempt (*M* = 50%, *SD* = 15%) and the second lowest was for disgust (*M* = 77.3%, *SD* = 11.1%) while highest agreement rate was for happy expression (*M* = 98%, *SD* = 3%). Since we did not include ‘contempt’ expression, we could not compare the two datasets for this particular expression.

A two-way RM ANOVA for agreement rates comparing the ratings from the Dutch and the Indian participants was performed with expression (7 expressions: happy, surprise, disgust, neutral, angry, sad, and fear) and culture (2 Cultures: Indian and Dutch) as within subjects factors. Mauchly’s test showed significant deviations from sphericity for the expression factor, W(6) = 0.13, *p <* .001. Greenhouse-Geisser corrections were applied to the expression factor (ε_Expression_ = 0.60). There was a significant main effect of expression, *F*(3.6, 136.8) = 25.90, *p <* .001, *η*_*p*_^*2*^ = 0.40, and culture, *F*(1, 38) = 29.90, *p <* .001, *η*_*p*_^*2*^ = 0.44. Also, the interaction between expression and culture was significant, *F*(6, 228) = 17.14, *p <* .001, *η*_*p*_^*2*^ = 0.31. Post-hoc Tukey Kramer’s analysis showed no significant difference (all *p* > .30) between agreement rates of Indian and Dutch raters for happy, surprise, disgust and sad expressions. But significantly low agreement rates were found among Indians compared to Dutch raters for angry (*p <* .01, *d* = 0.79), neutral (*p =* .045, *d* = 0.75) and fearful (*p <* .01, *d* = 1.36) expressions. As mentioned previously, angry and fear were the expressions for which lowest agreement rates were observed within Indian raters.

**Fig 4:**
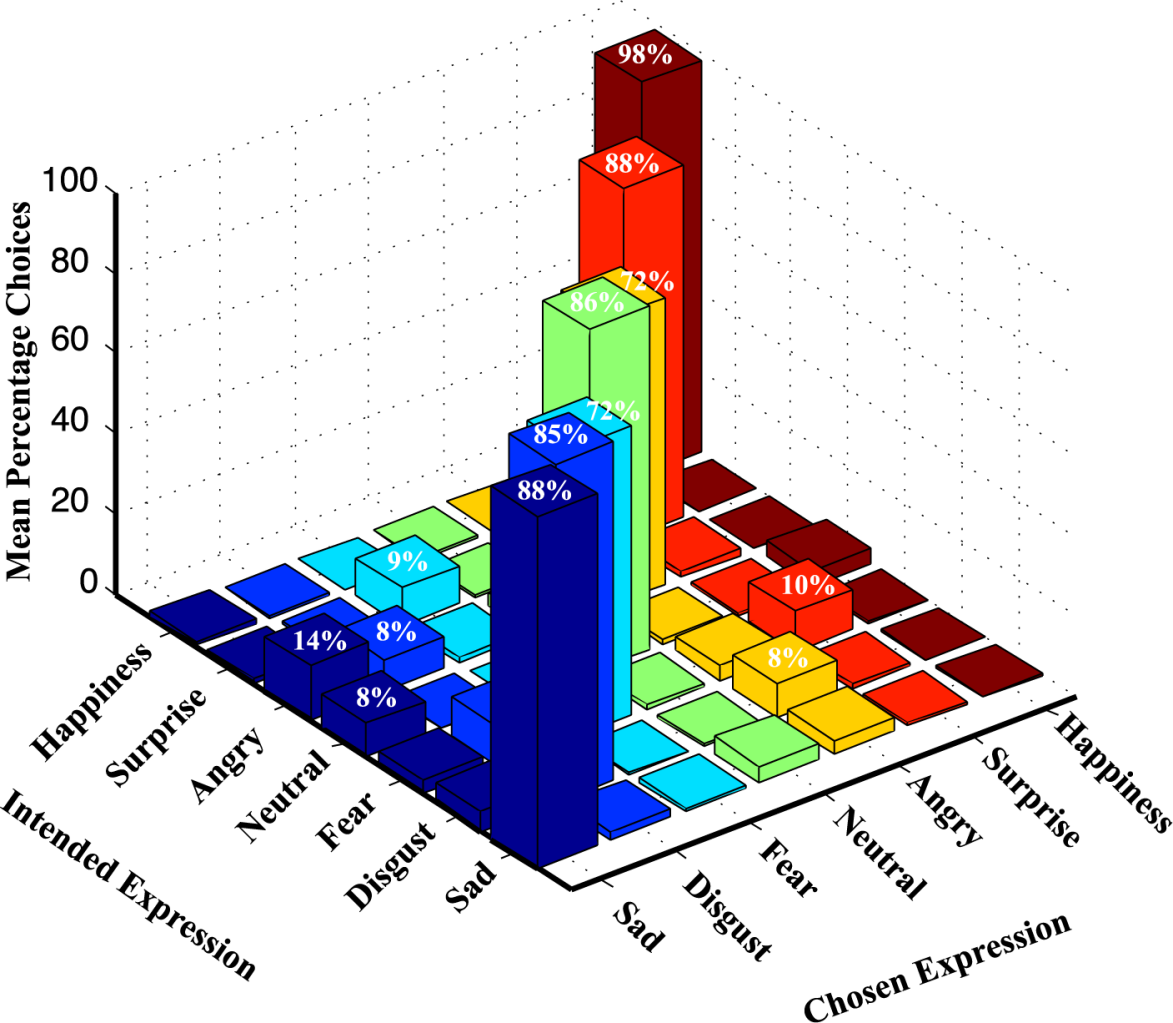
Unbiased hit rates. Three dimensional plot showing mean percentage (y-axis) of the chosen expression (z-axis) by Indian participants as a function of the intended expression (x-axis) of models in the Radboud database.

**Fig 5:**
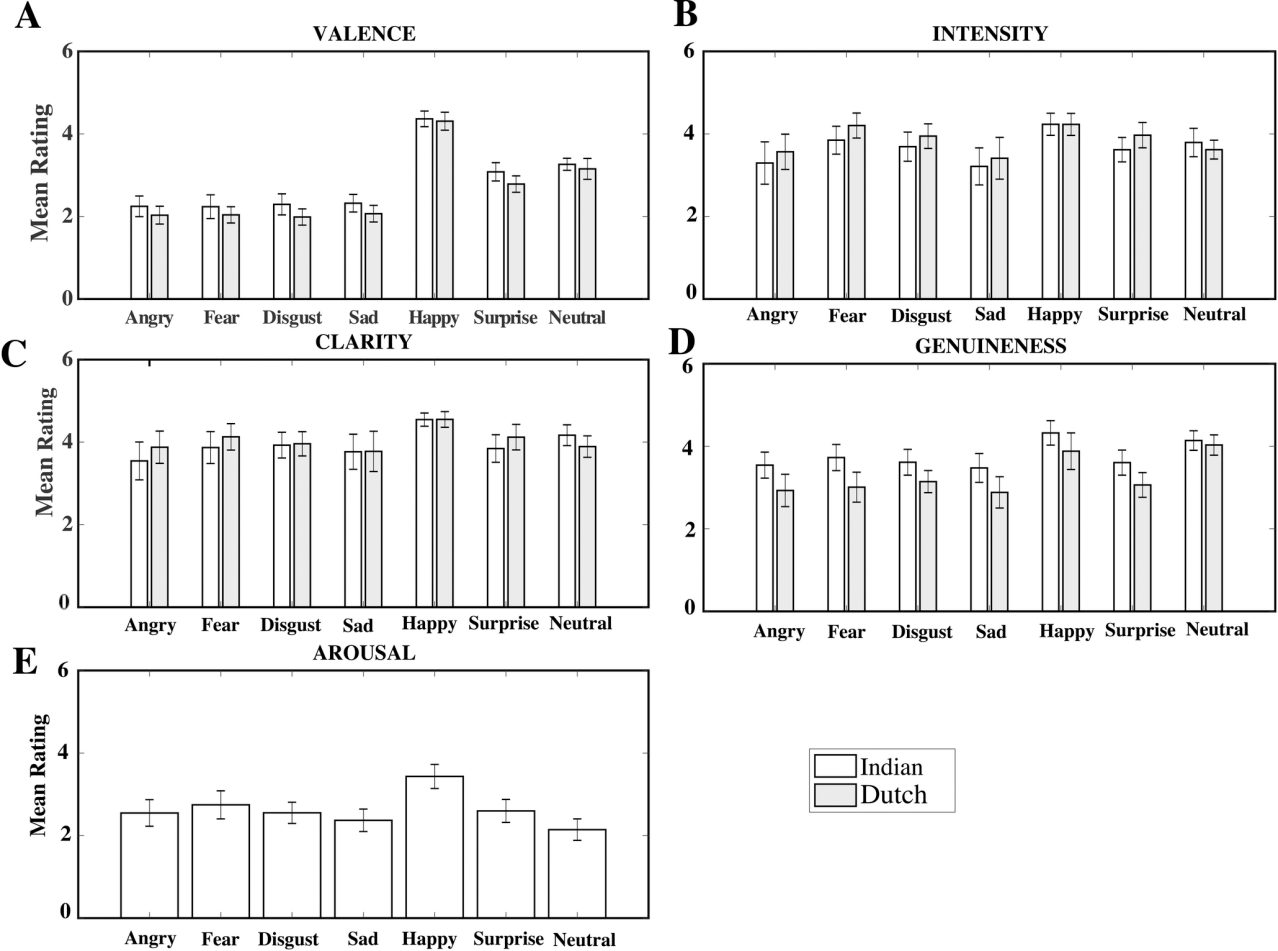
Histogram for valence, intensity, clarity, genuineness and arousal. Bar plots comparing mean ratings between Indian (white bars) and Dutch (grey bars) participants for valence (A), intensity (B), clarity (C), genuineness (D). For arousal (E) parameter mean ratings only for Indian participants is shown. The error bars represent standard error of mean.

There were few negative expressions (e.g. fear, angry) for which there was lack of consensus among raters and the agreement rates were low (~ 70%). Fig 4 shows a three-dimensional plot of mean percentage of chosen expressions by the participants, as a function of intended expressions by the models. This plot also represents a confusion matrix, that is, how often an intended expression (of a model in RaFD) was confused for any other expression in this force-choice paradigm. The confusion matrix shows that intended fear was confused as displaying surprise (10%) emotion, intended surprise was confused with fear (9%), and intended disgust was confused with angry (8%). Such categorization errors were also reported by the Dutch raters (see Fig 4, [5]). Indian raters categorized intended angry as sad (14%) and disgust (8%), while intended neutral was classified as sad (8%). Visual inspection indicates that Indian raters misclassified angry and neutral expressions more often as compared to Dutch raters.

### Unbiased hit rate analysis

To control for response bias (that is, the response key for a given emotion is used only for that emotion), we conducted an unbiased hit rate analysis using confusion matrix evaluated above [45] as reported by [5]. Low unbiased hit rates indicate that stimuli from a given category are not classified correctly.

Two-way RM ANOVA on arcsine transformed unbiased hit rates with expressions and gender of the models was performed with Greenhouse-Geisser correction for the expression (ε_Expression_ = 0.76) factor, as Mauchly’s test showed significant deviation from sphericity for the same (W(6) = 0.36, *p =* .009). Unlike Radboud analysis [5], there was no significant effect of gender of the model, *F*(1, 39) = 0.87, *p* = .36, in Indian ratings, but there was significant effect of expression, *F*(6, 234) = 44.79, *p <* .001, *η*_*p*_^*2*^ = 0.53. Post-hoc Tukey Kramer’s analysis showed that unbiased hit rate by Indian raters was higher for the happy expression (*M* = 93%, *SD* = 8.5%) compared to all other expressions; surprise, disgust, neutral, angry, fear, and sad (all *p <* .001, *all d* = 1.11 ≤ *d ≤ 2*.*24*). Hit-rates for disgust expression (*M* = 69.59%, *SD* = 18.42%) was significantly lower with respect to happy (*p <* .001, *d* = 2.1), fear (*p* = .048, *d* = 0.37), neutral (*p <* .001, *d* = 0.86), surprise (*p <* .001, *d* = 0.82) and angry (*p <* .001, *d* = 0.47). Hit-rate for disgust was not significantly different from sad (*p =* .95). The interaction between the gender of the model and the expression was not significant, *F*(6, 234) = 0.97, *p* = .45.

### Emotion ratings analysis

Fig 5 displays histograms for mean values of different rating parameters namely; valence (Fig 5A), intensity (Fig 5B), clarity (Fig 5C), and genuineness (Fig 5D) for Indian and Dutch raters and arousal (Fig 5E) for only Indian raters, across six expressions. To study the difference in rating parameters as a function of emotional facial expressions across the two cultures (Indians and Dutch), we performed a three way mixed ANOVA (2 x 2 x 7) for each individual rating parameter (valence, intensity, clarity, genuineness) with culture (Indian and Dutch) and expression (happy, surprise, disgust, neutral, angry, fear and sad) as within subjects factors and gender of the image (male and female) as a between subjects factor. For the arousal parameter since there were no corresponding ratings available from the Dutch participants, culture was not included as a parameter in the analysis.

#### Valence analysis

The main effect of culture, *F*(1, 37) = 94.90, *p* < .001, *η*_*p*_^*2*^ = 0.72 and expression, *F*(6, 222) = 985.90, *p* < .001, *η*_*p*_^*2*^ = 0.96 was significant. Mean valence rating for Indian raters (M = 2.83, SE = .018) were higher compared to Dutch raters (M = 2.62, SE = 0.17). As expected, happy expression was rated the most positive (*M* = 4.34, *SD* = 0.035). Negative emotions like disgust, angry, sad and fear had significantly (*p <* .01) low valence ratings compared to surprise, neutral, and happy expressions.

There was also a significant interaction between culture and expression, *F*(6, 222) = 4.75, *p* < .001, *η*_*p*_^*2*^ = 0.11. Post-hoc Tukey Kramer’s analysis showed that valence ratings were not significantly different between the two cultures for happy (*p =* .99), neutral (*p =* .08) and fear (*p =* .08) expressions. For all other expressions like surprise (*p <* .001, *d* = 1.6), disgust (*p <* .001, *d* = 1.40), angry (*p <* .01, *d* = 0.85) and sad (*p <* .01, *d* = 0.89), Indian ratings were significantly higher than Dutch ratings. The main effect of gender was not significant, *F*(1,37) = 0.015, *p =* .90. The interactions between expression and gender, *F*(6, 222) = 0.89, *p =* .50, culture and gender, *F*(1, 37) = 0.006, *p =* .94 and expression, culture and gender, *F*(6, 222) = 0.45, *p =* .81 were also not significant.

#### Intensity analysis

For intensity parameter, Greenhouse-Geisser correction was applied to within participant factor of Expression (ε_Expression_ = 0.69) as Mauchly’s test showed violation of sphericity (W(6) = 0.29, *p* < .001). The main effects of culture, *F*(1, 37) = 26.38, *p* < .001, *η*_*p*_^*2*^ = 0.41, and expression, *F*(4.14, 153.18) = 46.80, *p* < .001, *η*_*p*_^*2*^ = 0.55, were significant. Intensity ratings by Dutch raters were significantly higher than Indian raters (*p <* .001). Intensity rating for happy expression was significantly highest than all other expressions (*p <* .001). Similarly, intensity rating for fear expression was also significantly greater than all other expressions (*p <* .05), except happy. Ratings for happy expression were significantly higher than fear (*p =* .003).

The interaction between culture and expression was significant, *F*(6, 222) = 16.24, *p <* .001, *η*_*p*_^*2*^ = 0.30. Post-hoc Tukey Kramer’s analysis comparing effects of specific expressions across culture showed no significant difference in intensity ratings between Indian and Dutch raters for happy (*p* = 1.0), disgust (*p =* .82), fear (*p =* .05), neutral (*p =* .08) and sad (*p* = 0.59) expressions. Indian raters found surprise (*p <* .001, *d* = 1.19) and angry (*p =* .04, *d* = 0.65) expressions less intense than Dutch raters. In both cultures, happy expression was the most intense (*M* = 4.23) and sad was the least intense (*M* = ~3.4) expression. While, among Indian raters, happy was rated significantly higher than fear (*p <* .01, *d* = 1.12), the difference was not significant among Dutch raters (*p* > 0.05). There was no main effect of gender, *F*(1, 37) = 0.001, *p =* .97. The interactions between gender and expression, *F*(6, 222) = 1.96, *p =* .072, culture and gender *F*(1,37) = 1.67, *p =* .20, and three way interaction between expression, culture, and gender, *F*(6, 222) = 0.78, *p =* .58 was not significant.

#### Clarity analysis

For clarity parameter Mauchly’s test showed violation of sphericity for expression (ε_Expression_ = 0.66), gender X expression (ε_Expression X gender_ = 0.66) and culture X expression (ε_Expression X Culture_ = 0.76). The Greenhouse-Geisser corrected values for these factors: Expression, *W*(6) = 0.25, *p <* .001, gender X expression, *W*(6) = 0.25, *p <* .001, and culture X Expression, *W*(6) = 0.32, *p =* .007, were used in the analysis.

The main effect of culture was not significant, *F*(1, 37) = 0.67, *p =* .42; while the effect of expression was significant, *F*(3.96, 146.52) = 41.28, *p <* .001, *η*_*p*_^*2*^ = 0.52. Post-Hoc Tukey Kramer’s analysis showed that happy expression got significantly higher ratings (*M* = 4.54, *SD* = 0.17) than all other expressions (all *p <* .001, *all d* = 2.1 ≤ *d* ≤ 2.64). Angry was rated as the expression with least clarity and was significantly less than happy (*p <* .001, *d* = 2.56), disgust (*p <* .001, *d* = 0.54), neutral (*p <* .001, *d* = 0.81), fear (*p <* .001, *d* = 0.68) and surprise (*p <* .001, *d* = 0.70). Angry ratings were not significantly different than sad (*p =* .99).

The interaction between culture and expression, *F*(4.56,168.72) = 13.76, *p <* .001, *η*_*p*_^*2*^ = 0.27 was significant. Post-hoc Tukey-Kramer’s analysis for culture and expression showed that there were no significant differences in ratings between the two cultures for happy (*p* = 1.0), disgust (*p =* .99), angry (*p =* .056), fear (*p =* .55), surprise (*p =* .24) and sad (*p* = 1.0) expressions. The interaction effect was mainly due to the significantly low ratings in Dutch (*M* = 3.81, *SD* = 0.31) compared to Indian (*M* = 4.17, *SD* = 0.25) dataset for neutral expression (*p <* .001, *d* = 1.28). In both the datasets, highest clarity rating was for happy expression (*M* = 4.54, *SD* = 0.16; Dutch-*M* = 4.54, *SD* = 0.18) compared to all other six expressions. Lowest rating with Indian raters was observed for angry (*M* = 3.54, *D* = 0.46) expression. On the other hand, in Dutch raters, the lowest rating was observed for sad expression (*M* = 3.69, *SD* = 0.47).

The interaction between expression and gender, *F*(0.66, 146.52) = 2.78, *p =* .012, *η*_*p*_^*2*^ = 0.07 was also significant. Post-hoc analysis showed no significant difference in clarity ratings between male and female picture for happy (*p =* .57), surprise (*p =* .12), disgust (*p =* .16), neutral (*p =* .85), angry (*p =* .11), and sad (*p =* .05) expressions. But significantly high clarity rating (*p =* .02, *d* = 0.58) was observed for female (*M* = 4.06, *SD* = 0.38) models than male (*M* = 3.85, *SD* = 0.34) models portraying fear expression. The effects of gender, *F*(1, 37) = 0.66, *p =* .42, two way interaction between culture and gender, *F*(1,37) = 3.15, *p =* .084, and three way interaction between expression, culture, and gender, *F*(6, 222) = 0.81, *p =* .56, were not significant.

#### Genuineness analysis

The main effect of culture, *F*(1,37) = 395.36, *p* < .001, *η*_*p*_^*2*^ = 0.91, and expression, *F*(6,222) = 109.02, *p <* .001, *η*_*p*_^*2*^ = 0.74, was significant. Post-hoc Tukey Kramer’s analysis showed that the main effect of expression was attributed to overall significantly high genuineness ratings for happy and neutral than all other expressions, with no significant difference between the two (*p =* .99). Least rating was reported for sad expression, that was significantly less than happy (*p <* .001, *d* = 2.04), disgust (*p =* .008, *d* = 0.39), neutral (*p <* .001, *d* = 2.39), surprise (*p =* .02, *d* = 0.34) and fear (*p =* .001, *d* = 0.39). Genuineness ratings were significantly high among Indian (*M* = 3.26) compared to Dutch (*M* = 3.77) raters (*p <* .001, *d* = 1.17).

The interaction between culture and expression was significant, *F*(6, 222) = 10.11, *p <* .001, *η*_*p*_^*2*^ = 0.21. Post hoc Tukey Kramer’s analysis to study culture and expression interaction effects showed that there were no significant differences between Indian and Dutch ratings for neutral (*p =* .78) expression. On the other hand Indian ratings were significantly higher than Dutch for happy, angry, sad, surprise, disgust and fear (all *p <* .01, all *d* > 1.17). Highest rating for genuineness was given to happy expression by both Dutch (*M* = 3.88, *SD* = 0.44) and Indian (*M* = 4.32, *SD* = 0.29) participants, but these ratings were significantly higher in Indian than Dutch (*p* < .001, *d* = 1.17). Lowest ratings were observed for sad expressions in both Indian (*M* = 3.43, *SD* = 0.42) and Dutch (*M* = 2.90, *SD* = 0.34) datasets.

The interaction between gender and expression was significant, *F*(6, 222) = 3.87, *p =* .001, *η*_*p*_^*2*^ = 0.09. Post-hoc Tukey-Kramer analyses showed that ratings for angry males were higher than for angry females (*p =* .04, *d* = 0.48). On the other hand, female models had higher ratings than male models for sad (*p =* .04, *d* = 0.48) and fear (*p =* .03, *d* = 0.49) expressions. There were no significant differences between male and female models for happy (*p =* .40), surprise (*p =* .48), neutral (*p =* .99) and disgust (*p =* .25) expressions. The effects of gender, *F*(1, 37) = 2.56, *p =* .12, two way interaction between culture and gender, *F*(1, 37) = 1.06, *p =* .31, and three way interaction between culture, expression and gender were not significant, *F*(6, 222) = 0.90, *p =* .50.

#### Arousal analysis

Arousal analysis was conducted for Indian rating data only as there was no corresponding rating available in Dutch population. A two way mixed ANOVA with Gender of the models (2) as between subjects’ factor and Expression (7) as within subjects factor was performed.

There was a significant effect of expression, *F*(6, 222) = 66.77, *p <* .001, *η*_*p*_^*2*^ = 0.64; while the effect of gender, *F*(1, 37) = 2.21, *p =* .14, and interaction between gender and expression, *F*(6, 222) = 1.18, *p =* .31, were not significant. Post-hoc Tukey Kramer’s analysis showed that main effect of expression was attributed to significantly high arousal ratings for happy (*M* = 3.43, *SD* = 0.29) as compared to angry, surprise, fear, disgust, sad and neutral (all *p <* .001, *d* > 2.42) expressions. Lowest rating was given to neutral (*M* = 2.17, *SD* = 0.24) and was significantly different from happy, angry, surprise, fear and disgust, (all *p <* .001, all *d* > 3.0) expressions. However, arousal rating for neutral was not significantly different from sad (*p =* .22) expression.

### Correlation analysis

We also calculated Pearson’s correlations (Table 1) between different parameters and found significantly high correlations between intensity and clarity, *r*(270) = 0.67, *p <* .001. Similar high correlation between intensity and clarity was also reported amongst Dutch raters. With Dutch ratings [5], low correlations were observed for genuineness with intensity, *r* = 0.10 and clarity, *r* = 0.24. In contrast, genuineness was significantly correlated with clarity, *r*(270) = 0.66, *p <* .001, and intensity, *r*(270) = 0.58, *p <* .001, among Indian raters. Happy and Neutral expressions were rated as most genuine; also happy was the only expression rated as positive for valence. Neutral was rated as neutral valence (*M* = 3.2, *SD* = 0.18), and Surprise was rated close to neutral (*M* = 3.01, *SD* = 0.21).

**Table 1:** Correlation analysis across the five rating parameters.

	Valence	Intensity	Clarity	Genuineness	Arousal
Valence	NA	Ind = 0.37 p < .001	Ind = 0.54 p < .001	Ind = 0.53 p < .001	Ind = 0.37 p < .001
Intensity	NA	NA	Ind = 0.66 p < .001	Ind = 0.7 p < .001	Ind = 0.47 p < .001
Clarity	NA	NA	NA	Ind = 0.67 p < .001	Ind = 0.40 p < .001
Genuineness	NA	NA	NA	NA	Ind = 0.35 p < .001
Arousal	NA	NA	NA	NA	NA

### Inter-rater reliability index

In our study there are multiple models displaying similar emotions as well as each participant rates all the expression of any particular model. Thus, there is only one response from each participant/ emotion parameter. This gives each face 20 independent responses. In order to measure the strength/ consistency of ratings from these 20 different participants, Intra-class correlation coefficient (ICC) can be calculated. This analysis removes any measurement/ judgment errors given by the raters. ICC is also termed as an inter-rater reliability index, or as reliability coefficient [5,46]. ICC is the ratio between variance of the variable of interest with the sum of variance and error component. High values (near 1) indicate that the observations across various participants are similar; while values close to zero indicate that the participant’s responses differ and have lot of variability.

Required ICC for all the rating measures are presented in Table 2 and the values were similar to that from Dutch raters. As mentioned by Langner et al [5], we also could not parse between-rater variance out as we were not able to calculate higher indices ICC(2, 1) and ICC(2, k) due to the fact that different sets of participants rated different models. The values of ICC in our study are quite similar to Langner et al (2010), except that lesser values are observed for attractiveness, intensity and clarity. But still the values are closer to one rather than zero suggesting that there is similarity in the ratings of participants from India.

**Table 2:** Inter class correlations across five rating parameters.

Parameters	ICC (1,1)	ICC (1,k)
**Attractiveness**	**0.25**	**0.87**
**Intensity**	**0.15**	**0.78**
**Clarity**	**0.12**	**0.74**
**Genuineness**	**0.1**	**0.7**
**Valence**	**0.34**	**0.91**
**Arousal**	**0.09**	**0.66**

### Gender specific emotion analysis

Many studies have shown that there is a tendency of male faces to be rated angrier than female and similarly female faces to be rated happier than males. On the other hand, cross-cultural studies have also shown that participants rate faces from different culture/race angrier [47,48]. But this trend may depend upon whether a particular culture is individualistic or collectivistic. We wanted to test whether there are any cross-cultural differences in emotion recognition as a function of gender of the models; by raters from a collectivistic society like India (this study) and individualistic society like the Netherlands, for rating the same Caucasian model’s faces in both the cultures. For this analysis we used only valence and intensity parameters of happy and angry expressions.

To test if male models are rated angrier while female models are rated happier, we first performed Wilcoxon Rank sum test with Bonferroni correction (α = 0.01) between male and female images within Indian and Dutch rating data separately, for intensity and valence parameters. The test showed no significant difference in ratings of ‘valence’ and ‘intensity’ parameters between male and female images posing happy and angry expressions for both the populations (*p* > .05). These results do not support gender-based expression ratings [47,48] for happy and angry expressions, at least with the simple emotion perception and rating task used in this study.

## Discussion

Using within-culture and cross-cultural analysis, this study has addressed few of the central questions in facial emotion recognition in the context of culture about; a) universality of emotion recognition across cultures, b) contribution of specific features of emotional faces (other than agreement rates) in evaluating universality, c) differences in emotion ratings by individualistic and collectivistic societies, and d) validation of the Radboud database to be used in an appropriate manner by Indian participants in future studies. In order to achieve this, we evaluated the emotion categorization accuracy (agreement rates), valence, intensity, clarity, genuineness and arousal judgments by Indian raters, employing a similar design and methodology as the original study [5] and comparing it with already available performance and ratings from Dutch raters. This also enabled us to validate the Radboud database, so that it can be used in the Indian context for the Indian population.

*Are emotions classified universally across different cultures*? An important measure to test universality is by investigating the recognition accuracy (agreement rate measurements) across different facial expressions. We observed that the overall agreement rate provided by Indian participants across all emotion categories (88%) was comparable (88%) to that of Dutch participants. When this overall agreement data was divided into individual emotion categories, happy faces were most correctly recognized in comparison to all other expressions. This was evident by significantly higher values for mean agreement rates, unbiased hit rates as well as for the parameters of valence, intensity, clarity and genuineness for happy expression.

A more critical way to understand universality in emotion recognition performance is through the confusion matrix or unbiased hit rates analysis. The Indian ratings again show that least confusion was observed for happy emotion recognition than all other emotional categories. This is also in line with the ratings by Dutch participants and implies that ‘happy’ can be considered as the least ambiguous expression in the Radboud database across both the cultures. Angry and fear had the lowest agreement rates and high misclassifications among Indian raters. However, with the Dutch raters, contempt and disgust were rated lowest and highly misclassified. It should be noted that we did not use the contempt expression in our study. For other expressions (happy, neutral, sad and surprise), the mean agreement rates were comparable between Dutch and Indian raters (see, S1 Table).

The above results for happy expression are supported by other database validation and cross cultural studies. The main differences in misclassifications were seen with high arousal negative expressions for both cultures, consistent with findings reported in cross-culture emotion studies [3,30,49,50]. Thus, as far as recognition accuracy is considered, happy expression can be said as to be recognized more universally. The observations are in line with the literature suggesting that Universality hypothesis may hold true [30,49,51], but only for specific emotion categories among cultural groups. A closer look at misclassification errors indicates significant differences across the two cultures; suggesting cultural differences and thus argues against strict universality.

In recent years, many studies have emphasized the use of measuring various parameters that affect the quality of perceived emotion. These include valence, intensity, clarity, genuineness, arousal as well as others (like trustworthiness). These parameters help in establishing the strength of emotion processing as well as a control for various experimental studies where parameters such as valence and arousal play differential roles in studies on emotion-attention interactions [32–38]. The results obtained by comparing specific emotional parameters across the two cultures revealed that all parameters (except clarity of emotion) were significantly different between cultures and showed significant interactions between culture and expression. Specifically, for valence and genuineness parameters, Indians rated specific emotions (happy, angry, sad, surprise, disgust and fear) as more positive or negative and more genuine than the Dutch participants. In contrast, the intensity of few emotions (surprise and angry) were rated higher by Dutch participants than Indians. The expression with most similar ratings for valence, intensity and clarity was the happy expression. Even with happy expression, Indian raters rated it as more genuine and more arousing. These results indicate that even though the participants are able to correctly classify/categorize emotions across culture, there are significant parameter specific differences in emotion perception that do not fully support the universality hypothesis between the two cultures.

As discussed in the introduction, a significant factor that may contribute to such cultural differences may reside within the behavior of societies; that is whether a particular culture is more individualistic or collectivistic [21,24,26]. Very few studies have evaluated this aspect in the context of perception of emotional faces rated by Indian participants. The current study compares ratings of out-group faces (Caucasian) by Indian raters and in-group faces by Dutch raters. For example, the agreement rates by Indian participants for negative emotions like anger and fear were significantly lower than those reported by Dutch participants (Fig 3). Additionally high misclassification errors were observed with Indian raters for negative emotions. This is consistent with studies that show better accuracy with individualistic compared to collectivistic societies [22,29,30]. Low accuracy for out-group negative emotions in a collectivistic society has been attributed to the fact that these emotions are discouraged in the context of interdependence and group formation [24,29]. Further, the mean intensity ratings of Indian participants were in general less than Dutch participants for most emotions (Fig 5B), but ratings for only angry and surprise showed statistical significance. This is partly consistent with results showing high intensity ratings with individualistic compared to collectivistic societies [21,31].

The results from the current study and inferences made on the basis of individualistic and collectivistic societies is in contrast to an earlier study [18], which compared valence ratings across three cultures (Indian, Japanese and American) with models posing for seven basic emotions also being from above three cultures. They did not observe biases in valence ratings depending upon whether a culture is individualistic or collectivist. One reason could be that the models posing for emotions in Indian and Japanese culture did not follow the FACS guidelines. The failure to follow FACS guidelines was used to explain the significantly high accuracy rates for American models posing for different emotions compared to the models from the other two cultures [18] and may have resulted in lack of differences in valence ratings across cultures. Further, highest accuracy was observed for participants performing emotion recognition from the same culture, referred to as the ‘in-group advantage [17,43]. This in-group advantage is known to be reduced as a function of geographical proximity and cross-cultural interactions amongst the cultural groups tested [22,52,53,54]. This is evident in our study as well; for the expressions with significant difference between the two cultures, the agreement rates were higher in Dutch than the Indian data-set.

It has been argued that the in-group advantage disappears when models use a standardized protocol like FACS [2] for portraying emotions [22]. It is suggested that emotion stimuli database developed following FACS notation augments ‘stimulus equivalence’, but at the same time due to difference in level of intensity of portraying emotions by different models and due to physiognomic differences (encoder effect), the decoding would be affected to different extent across cultures, leading to cross-cultural variations (decoder effect) in the emotion recognition [22]. This is confirmed in our study also by misclassification analysis, which showed pronounced differences across the two cultures. The misclassification errors for Indian raters were more for all the expressions except happy and sad. On the other hand misclassification errors by Dutch raters were relatively less and restricted to surprise, fear, disgust and contempt. These results raise questions about universality of emotion perception and point to subtle cross-cultural differences in emotion perception.

Pertaining to gender differences in emotional expressions, within Indian and Dutch raters, we did not observe any significant difference in valence or intensity ratings between male and female face models posing happy and angry emotions. However, we did observe significant effect of model gender on clarity and genuineness parameters, but these were limited to negative valence emotions only (fear, sad and anger). Moreover, there was no significant interaction between culture and gender for any of the four parameters tested. These results do not support (at least with Indians rating the Radboud faces) the idea that males from other cultures are rated angrier, while female are rated happier [47,48].

The current study is at the core of an existing debate on universality of emotion recognition across different cultures. There is literature in support [12,15,51], and against [14,18,52] the universality theory. However, a general consensus is that emotion recognition across cultures for the basic emotions (happy, angry, sad, disgust, fear, surprise) are above chance level and are recognized reliably, but the accuracy varies across cultures [18,21,53]. This difference in accuracy has been attributed to various factors such as subtle differences in the expression style of different facial emotions across different cultures [19], familiarity of an emotion within a culture [52] or the frequency of occurrence of an emotion in a cultural group [30]. Our study reports that out of all the emotions used, ratings for happy recognition is most consistent across the two cultures, while significant differences in culture exist among other emotional categories and more specific features of emotional faces, arguing against the universality of emotion perception.

## Conclusion

In conclusion, we would like to emphasize the importance of validation of image databases for studies on emotion processing in different cultures. This study facilitates the use of an established database like the Radboud database in India. While advocating the use of a cross-cultural database, caution must be exercised since not all expressions are classified or rated in the same manner compared to the original ratings. From a theoretical perspective, the study not only indicates cross-cultural differences in emotion classification but also demonstrates the presence of subtle differences in emotion perception even when an emotion is accurately categorized, raising questions about the universality of emotion perception.

## Supporting information

S1 Table Hit rates (mean values) for individual picture of Radboud database by Indian and Dutch raters. (DOCX)

## References

[1] Izard CE. Maximally discriminative facial movement coding system. University of Delaware, instructional resources Center; 1979.

[2] Ekman P, Friesen W V, Hager JC. Facial action coding system (FACS). A Tech Meas facial action Consult Palo Alto. 1978;22.

[3] Tottenham N, Tanaka JW, Leon AC, McCarry T, Nurse M, Hare TA, et al The NimStim set of facial expressions: Judgments from untrained research participants. Psychiatry Res [Internet]. 2009;168(3):242–9. Available from: 10.1016/j.psychres.2008.05.006 19564050 PMC3474329

[4] Lundqvist D, Flykt A, Öhman A. The Karolinska directed emotional faces (KDEF). CD ROM from Dep Clin Neurosci Psychol Sect Karolinska Institutet 1998;91–630.

[5] Langner O, Dotsch R, Bijlstra G, Wigboldus DHJ, Hawk ST, van Knippenberg A. Presentation and validation of the Radboud Faces Database. Cogn Emot. 2010;24(8):1377–88.

[6] Ekman P, Friesen W V, Ellsworth P. Emotion in the Human Face: Guide-lines for Research and an Integration of Findings: Guidelines for Research and an Integration of Findings. Pergamon; 1972.

[7] Fridlund AJ, Ekman P, Oster H. Facial expressions of emotion. 1987;

[8] Ekman P. Strong evidence for universals in facial expressions: a reply to Russell’s mistaken critique. 1994; 10.1037/0033-2909.115.2.268 8165272

[9] Gendron M, Roberson D, van der Vyver JM, Barrett LF. Perceptions of emotion from facial expressions are not culturally universal: evidence from a remote culture. Emotion. 2014;14(2):251 10.1037/a0036052 24708506 PMC4752367

[10] Hwang H, Matsumoto D. Evidence for the universality of facial expressions of emotion In: Understanding Facial Expressions in Communication. Springer; 2015 p. 41–56.

[11] Nelson NL, Russell JA. Universality revisited. Emot Rev. 2013;5(1):8–15.

[12] Ekman P, Friesen W V. Constants across cultures in the face and emotion. J Pers Soc Psychol. 1971;17(2):124 5542557 10.1037/h0030377

[13] Yan X, Andrews TJ, Young AW. Cultural similarities and differences in perceiving and recognizing facial expressions of basic emotions. J Exp Psychol Hum Percept Perform. 2016;42(3):423 10.1037/xhp0000114 26480247

[14] Jack RE, Garrod OGB, Yu H, Caldara R, Schyns PG. Facial expressions of emotion are not culturally universal. Proc Natl Acad Sci. 2012;109(19):7241–4. 10.1073/pnas.1200155109 22509011 PMC3358835

[15] Ekman P, Sorenson ER, Friesen W V. Pan-cultural elements in facial displays of emotion. Science (80-). 1969;164(3875):86–8. 5773719 10.1126/science.164.3875.86

[16] Russell JA. Is there universal recognition of emotion from facial expressions? A review of the cross-cultural studies. Psychol Bull. 1994;115(1):102 8202574 10.1037/0033-2909.115.1.102

[17] Elfenbein HA, Ambady N. Is there an in-group advantage in emotion recognition? 2002; 10.1037/0033-2909.128.2.243 11931518

[18] Elfenbein HA, Mandal MK, Ambady N, Harizuka S, Kumar S. Cross-cultural patterns in emotion recognition: highlighting design and analytical techniques. Emotion. 2002;2(1):75 12899367 10.1037/1528-3542.2.1.75

[19] Elfenbein HA, Ambady N. On the universality and cultural specificity of emotion recognition: a meta-analysis. Psychol Bull. 2002;128(2):203 11931516 10.1037/0033-2909.128.2.203

[20] Jack RE, Caldara R, Schyns PG. Internal representations reveal cultural diversity in expectations of facial expressions of emotion. J Exp Psychol Gen [Internet]. 2012;141(1):19–25. Available from: 10.1037/a0023463%5Cnhttp://doi.apa.org/getdoi.cfm?doi=10.1037/a0023463 21517206

[21] Matsumoto D. Cultural similarities and differences in display rules. Motiv Emot. 1990;14(3):195–214.

[22] Beaupré MG, Hess U. Cross-cultural emotion recognition among Canadian ethnic groups. J Cross Cult Psychol. 2005;36(3):355–70.

[23] Stephan WG, Stephan CW, De Vargas MC. Emotional expression in Costa Rica and the United States. J Cross Cult Psychol. 1996;27(2):147–60.

[24] Matsumoto D, Yoo SH, Fontaine J. Mapping expressive differences around the world: The relationship between emotional display rules and individualism versus collectivism. J Cross Cult Psychol. 2008;39(1):55–74.

[25] Kim UE, Triandis HC, Kâğitçibaşi ÇE, Choi S-CE, Yoon GE. Individualism and collectivism: Theory, method, and applications. Sage Publications, Inc; 1994.

[26] Oyserman D, Coon HM, Kemmelmeier M. Rethinking individualism and collectivism: evaluation of theoretical assumptions and meta-analyses. American Psychological Association; 2002. 11843547

[27] Sinha D, Tripathi RC. Individualism in a collectivist culture: A case of coexistence of opposites. 1994;

[28] Sinha JBP. Psycho-social analysis of the Indian mindset. Springer; 2014.

[29] Matsumoto D. American-Japanese cultural differences in the recognition of universal facial expressions. J Cross Cult Psychol. 1992;23(1):72–84.

[30] Biehl M, Matsumoto D, Ekman P, Hearn V, Heider K, Kudoh T, et al Matsumoto and Ekman’s Japanese and Caucasian Facial Expressions of Emotion (JACFEE): Reliability data and cross-national differences. J Nonverbal Behav. 1997;21(1):3–21.

[31] Matsumoto D. Cultural influences on the perception of emotion. J Cross Cult Psychol. 1989;20(1):92–105.

[32] Young AW, Rowland D, Calder AJ, Etcoff NL, Seth A, Perrett DI. Facial expression megamix: Tests of dimensional and category accounts of emotion recognition. Cognition. 1997;63(3):271–313. 9265872 10.1016/s0010-0277(97)00003-6

[33] Posner J, Russell JA, Peterson BS. The circumplex model of affect: An integrative approach to affective neuroscience, cognitive development, and psychopathology. Dev Psychopathol. 2005;17(3):715–34. 10.1017/S0954579405050340 16262989 PMC2367156

[34] Anderson AK. Affective Influences on the Attentional Dynamics Supporting Awareness. 2005;134(2):258–81. 10.1037/0096-3445.134.2.258 15869349

[35] Ihssen N, Keil A. The costs and benefits of processing emotional stimuli during rapid serial visual presentation. Cogn Emot. 2009;23(2):296–326.

[36] Fernandes M a., Koji S, Dixon MJ, Aquino JM. Changing the focus of attention: The interacting effect of valence and arousal. Vis cogn. 2011;19(9):1191–211.

[37] Jefferies LN, Smilek D, Eich E, Enns JT. Emotional Valence and Arousal Interact in Attentional Control. 2008;19(3):290–5. 10.1111/j.1467-9280.2008.02082.x 18315803

[38] Lundqvist D, Juth P, Öhman A. Using facial emotional stimuli in visual search experiments: The arousal factor explains contradictory results. Cogn Emot. 2014;28(6):1012–29. 10.1080/02699931.2013.867479 24341823

[39] Schneider W, Eschman A, Zuccolotto A. E-Prime reference guide. Psychology Software Tools, Incorporated; 2002.

[40] Matlab2012b. Natick, MA: MathWorks; 2012.

[41] R Core Team. R: A language and environment for statistical computing. Vienna, Austria: R Foundation for Statistical Computing; 2016 Available from: https://www.r-project.org/

[42] Matsumoto D, Ekman P. The relationship among expressions, labels, and descriptions of contempt. J Pers Soc Psychol. 2004;87(4):529 10.1037/0022-3514.87.4.529 15491276

[43] Elfenbein HA, Beaupré M, Lévesque M, Hess U. Toward a dialect theory: cultural differences in the expression and recognition of posed facial expressions. Emotion. 2007;7(1):131 10.1037/1528-3542.7.1.131 17352569

[44] Matsumoto D, Consolacion T, Yamada H, Suzuki R, Franklin B, Paul S, et al American-Japanese cultural differences in judgements of emotional expressions of different intensities. Cogn Emot. 2002;16(6):721–47.

[45] Wagner HL. On measuring performance in category judgment studies of nonverbal behavior. J Nonverbal Behav. 1993;17(1):3–28. Available from: http://link.springer.com/10.1007/BF00987006

[46] Shrout PE, Fleiss JL. Intraclass correlations: uses in assessing rater reliability. Psychol Bull. 1979;86(2):420 18839484 10.1037//0033-2909.86.2.420

[47] Becker DV, Kenrick DT, Neuberg SL, Blackwell KC, Smith DM. The confounded nature of angry men and happy women. J Pers Soc Psychol. 2007;92(2):179. 17279844 10.1037/0022-3514.92.2.179

[48] Hess U, Blairy S, Kleck RE. The intensity of emotional facial expressions and decoding accuracy. J Nonverbal Behav. 1997;21(4):241–57.

[49] Samuelsson H, Jarnvik K, Henningsson H, Andersson J, Carlbring P. The Umeå university database of facial expressions: a validation study. J Med Internet Res. 2012;14(5):e136 10.2196/jmir.2196 23047935 PMC3510711

[50] Goeleven E, De Raedt R, Leyman L, Verschuere B. The Karolinska directed emotional faces: a validation study. Cogn Emot. 2008;22(6):1094–118.

[51] Ekman P, Friesen W V. A new pan-cultural facial expression of emotion. Motiv Emot. 1986;10(2):159–68.

[52] Elfenbein HA, Ambady N. When familiarity breeds accuracy: cultural exposure and facial emotion recognition. J Pers Soc Psychol. 2003;85(2):276 12916570 10.1037/0022-3514.85.2.276

[53] Matsumoto D. Methodological requirements to test a possible in-group advantage in judging emotions across cultures: comment on Elfenbein and Ambady (2002) and evidence. 2002; 11931517 10.1037/0033-2909.128.2.236

[54] Prado C, Mellor D, Byrne LK, Wilson C, Xu X, Liu H. Facial emotion recognition: a cross-cultural comparison of Chinese, Chinese living in Australia, and Anglo-Australians. Motiv Emot. 2014;38(3):420–8.

