# Supplementary material for: Cross-cultural emotion recognition and evaluation of Radboud faces database with an Indian sample
Source: PLoS One. 2018 Oct 1;13(10):e0203959. doi: 10.1371/journal.pone.0203959 (PMC6166925; doi:10.1371/journal.pone.0203959)
Supplement: S1 Table — (DOCX) [file pone.0203959.s001.docx]

**Supporting information**

**S1 Table. Hit rates for individual picture of Radboud database by Indian and Dutch raters.**

| S No. | Name of the image | Indian agreement  (%) | Radboud agreement (%) |
| --- | --- | --- | --- |
| \|  \| \| --- \| \| 1 \| \| 2 \| \| 3 \| \| 4 \| \| 5 \| \| 6 \| \| 7 \| \| 8 \| \| 9 \| \| 10 \| \| 11 \| \| 12 \| \| 13 \| \| 14 \| \| 15 \| \| 16 \| \| 17 \| \| 18 \| \| 19 \| \| 20 \| \| 21 \| \| 22 \| \| 23 \| \| 24 \| \| 25 \| \| 26 \| \| 27 \| \| 28 \| \| 29 \| \| 30 \| \| 31 \| \| 32 \| \| 33 \| \| 34 \| \| 35 \| \| 36 \| \| 37 \| \| 38 \| \| 39 \| \| 40 \| \| 41 \| \| 42 \| \| 43 \| \| 44 \| \| 45 \| \| 46 \| \| 47 \| \| 48 \| \| 49 \| \| 50 \| \| 51 \| \| 52 \| \| 53 \| \| 54 \| \| 55 \| \| 56 \| \| 57 \| \| 58 \| \| 59 \| \| 60 \| \| 61 \| \| 62 \| \| 63 \| \| 64 \| \| 65 \| \| 66 \| \| 67 \| \| 68 \| \| 69 \| \| 70 \| \| 71 \| \| 72 \| \| 73 \| \| 74 \| \| 75 \| \| 76 \| \| 77 \| \| 78 \| \| 79 \| \| 80 \| \| 81 \| \| 82 \| \| 83 \| \| 84 \| \| 85 \| \| 86 \| \| 87 \| \| 88 \| \| 89 \| \| 90 \| \| 91 \| \| 92 \| \| 93 \| \| 94 \| \| 95 \| \| 96 \| \| 97 \| \| 98 \| \| 99 \| \| 100 \| \| 101 \| \| 102 \| \| 103 \| \| 104 \| \| 105 \| \| 106 \| \| 107 \| \| 108 \| \| 109 \| \| 110 \| \| 111 \| \| 112 \| \| 113 \| \| 114 \| \| 115 \| \| 116 \| \| 117 \| \| 118 \| \| 119 \| \| 120 \| \| 121 \| \| 122 \| \| 123 \| \| 124 \| \| 125 \| \| 126 \| \| 127 \| \| 128 \| \| 129 \| \| 130 \| \| 131 \| \| 132 \| \| 133 \| \| 134 \| \| 135 \| \| 136 \| \| 137 \| \| 138 \| \| 139 \| \| 140 \| \| 141 \| \| 142 \| \| 143 \| \| 144 \| \| 145 \| \| 146 \| \| 147 \| \| 148 \| \| 149 \| \| 150 \| \| 151 \| \| 152 \| \| 153 \| \| 154 \| \| 155 \| \| 156 \| \| 157 \| \| 158 \| \| 159 \| \| 160 \| \| 161 \| \| 162 \| \| 163 \| \| 164 \| \| 165 \| \| 166 \| \| 167 \| \| 168 \| \| 169 \| \| 170 \| \| 171 \| \| 172 \| \| 173 \| \| 174 \| \| 175 \| \| 176 \| \| 177 \| \| 178 \| \| 179 \| \| 180 \| \| 181 \| \| 182 \| \| 183 \| \| 184 \| \| 185 \| \| 186 \| \| 187 \| \| 188 \| \| 189 \| \| 190 \| \| 191 \| \| 192 \| \| 193 \| \| 194 \| \| 195 \| \| 196 \| \| 197 \| \| 198 \| \| 199 \| \| 200 \| \| 201 \| \| 202 \| \| 203 \| \| 204 \| \| 205 \| \| 206 \| \| 207 \| \| 208 \| \| 209 \| \| 210 \| \| 211 \| \| 212 \| \| 213 \| \| 214 \| \| 215 \| \| 216 \| \| 217 \| \| 218 \| \| 219 \| \| 220 \| \| 221 \| \| 222 \| \| 223 \| \| 224 \| \| 225 \| \| 226 \| \| 227 \| \| 228 \| \| 229 \| \| 230 \| \| 231 \| \| 232 \| \| 233 \| \| 234 \| \| 235 \| \| 236 \| \| 237 \| \| 238 \| \| 239 \| \| 240 \| \| 241 \| \| 242 \| \| 243 \| \| 244 \| \| 245 \| \| 246 \| \| 247 \| \| 248 \| \| 249 \| \| 250 \| \| 251 \| \| 252 \| \| 253 \| \| 254 \| \| 255 \| \| 256 \| \| 257 \| \| 258 \| \| 259 \| \| 260 \| \| 261 \| \| 262 \| \| 263 \| \| 264 \| \| 265 \| \| 266 \| \| 267 \| \| 268 \| \| 269 \| \| 270 \| \| 271 \| \| 272 \| \| 273 \| | \|  \| \| --- \| \| Rafd090_15_Caucasian_male_sad_frontal \| \| Rafd090_61_Caucasian_female_angry_frontal \| \| Rafd090_26_Caucasian_female_angry_frontal \| \| Rafd090_24_Caucasian_male_sad_frontal \| \| Rafd090_10_Caucasian_male_angry_frontal \| \| Rafd090_47_Caucasian_male_fearful_frontal \| \| Rafd090_16_Caucasian_female_angry_frontal \| \| Rafd090_18_Caucasian_female_angry_frontal \| \| Rafd090_09_Caucasian_male_disgusted_frontal \| \| Rafd090_56_Caucasian_female_angry_frontal \| \| Rafd090_30_Caucasian_male_fearful_frontal \| \| Rafd090_38_Caucasian_male_angry_frontal \| \| Rafd090_47_Caucasian_male_angry_frontal \| \| Rafd090_05_Caucasian_male_fearful_frontal \| \| Rafd090_07_Caucasian_male_angry_frontal \| \| Rafd090_32_Caucasian_female_fearful_frontal \| \| Rafd090_19_Caucasian_female_angry_frontal \| \| Rafd090_07_Caucasian_male_fearful_frontal \| \| Rafd090_22_Caucasian_female_angry_frontal \| \| Rafd090_37_Caucasian_female_fearful_frontal \| \| Rafd090_56_Caucasian_female_neutral_frontal \| \| Rafd090_58_Caucasian_female_fearful_frontal \| \| Rafd090_30_Caucasian_male_disgusted_frontal \| \| Rafd090_33_Caucasian_male_angry_frontal \| \| Rafd090_33_Caucasian_male_fearful_frontal \| \| Rafd090_16_Caucasian_female_neutral_frontal \| \| Rafd090_57_Caucasian_female_fearful_frontal \| \| Rafd090_09_Caucasian_male_surprised_frontal \| \| Rafd090_10_Caucasian_male_fearful_frontal \| \| Rafd090_31_Caucasian_female_fearful_frontal \| \| Rafd090_37_Caucasian_female_surprised_frontal \| \| Rafd090_24_Caucasian_male_disgusted_frontal \| \| Rafd090_36_Caucasian_male_fearful_frontal \| \| Rafd090_49_Caucasian_male_fearful_frontal \| \| Rafd090_19_Caucasian_female_surprised_frontal \| \| Rafd090_20_Caucasian_male_fearful_frontal \| \| Rafd090_20_Caucasian_male_neutral_frontal \| \| Rafd090_21_Caucasian_male_fearful_frontal \| \| Rafd090_57_Caucasian_female_neutral_frontal \| \| Rafd090_03_Caucasian_male_angry_frontal \| \| Rafd090_04_Caucasian_female_fearful_frontal \| \| Rafd090_08_Caucasian_female_fearful_frontal \| \| Rafd090_26_Caucasian_female_fearful_frontal \| \| Rafd090_27_Caucasian_female_angry_frontal \| \| Rafd090_49_Caucasian_male_angry_frontal \| \| Rafd090_12_Caucasian_female_angry_frontal \| \| Rafd090_12_Caucasian_female_fearful_frontal \| \| Rafd090_15_Caucasian_male_fearful_frontal \| \| Rafd090_16_Caucasian_female_disgusted_frontal \| \| Rafd090_02_Caucasian_female_fearful_frontal \| \| Rafd090_03_Caucasian_male_neutral_frontal \| \| Rafd090_04_Caucasian_female_angry_frontal \| \| Rafd090_05_Caucasian_male_surprised_frontal \| \| Rafd090_09_Caucasian_male_fearful_frontal \| \| Rafd090_32_Caucasian_female_disgusted_frontal \| \| Rafd090_58_Caucasian_female_disgusted_frontal \| \| Rafd090_61_Caucasian_female_neutral_frontal \| \| Rafd090_23_Caucasian_male_fearful_frontal \| \| Rafd090_24_Caucasian_male_fearful_frontal \| \| Rafd090_24_Caucasian_male_surprised_frontal \| \| Rafd090_28_Caucasian_male_angry_frontal \| \| Rafd090_28_Caucasian_male_disgusted_frontal \| \| Rafd090_28_Caucasian_male_surprised_frontal \| \| Rafd090_30_Caucasian_male_sad_frontal \| \| Rafd090_71_Caucasian_male_angry_frontal \| \| Rafd090_14_Caucasian_female_angry_frontal \| \| Rafd090_18_Caucasian_female_fearful_frontal \| \| Rafd090_20_Caucasian_male_angry_frontal \| \| Rafd090_21_Caucasian_male_sad_frontal \| \| Rafd090_57_Caucasian_female_angry_frontal \| \| Rafd090_02_Caucasian_female_neutral_frontal \| \| Rafd090_04_Caucasian_female_neutral_frontal \| \| Rafd090_07_Caucasian_male_sad_frontal \| \| Rafd090_07_Caucasian_male_surprised_frontal \| \| Rafd090_08_Caucasian_female_angry_frontal \| \| Rafd090_10_Caucasian_male_disgusted_frontal \| \| Rafd090_10_Caucasian_male_neutral_frontal \| \| Rafd090_27_Caucasian_female_disgusted_frontal \| \| Rafd090_37_Caucasian_female_disgusted_frontal \| \| Rafd090_56_Caucasian_female_fearful_frontal \| \| Rafd090_56_Caucasian_female_surprised_frontal \| \| Rafd090_24_Caucasian_male_angry_frontal \| \| Rafd090_25_Caucasian_male_angry_frontal \| \| Rafd090_25_Caucasian_male_disgusted_frontal \| \| Rafd090_25_Caucasian_male_fearful_frontal \| \| Rafd090_25_Caucasian_male_neutral_frontal \| \| Rafd090_36_Caucasian_male_disgusted_frontal \| \| Rafd090_36_Caucasian_male_neutral_frontal \| \| Rafd090_38_Caucasian_male_fearful_frontal \| \| Rafd090_46_Caucasian_male_surprised_frontal \| \| Rafd090_47_Caucasian_male_neutral_frontal \| \| Rafd090_12_Caucasian_female_sad_frontal \| \| Rafd090_14_Caucasian_female_disgusted_frontal \| \| Rafd090_14_Caucasian_female_neutral_frontal \| \| Rafd090_14_Caucasian_female_sad_frontal \| \| Rafd090_15_Caucasian_male_angry_frontal \| \| Rafd090_16_Caucasian_female_fearful_frontal \| \| Rafd090_18_Caucasian_female_neutral_frontal \| \| Rafd090_18_Caucasian_female_surprised_frontal \| \| Rafd090_19_Caucasian_female_fearful_frontal \| \| Rafd090_19_Caucasian_female_sad_frontal \| \| Rafd090_21_Caucasian_male_angry_frontal \| \| Rafd090_21_Caucasian_male_disgusted_frontal \| \| Rafd090_21_Caucasian_male_surprised_frontal \| \| Rafd090_01_Caucasian_female_fearful_frontal \| \| Rafd090_02_Caucasian_female_sad_frontal \| \| Rafd090_03_Caucasian_male_fearful_frontal \| \| Rafd090_05_Caucasian_male_disgusted_frontal \| \| Rafd090_07_Caucasian_male_disgusted_frontal \| \| Rafd090_08_Caucasian_female_sad_frontal \| \| Rafd090_09_Caucasian_male_neutral_frontal \| \| Rafd090_09_Caucasian_male_sad_frontal \| \| Rafd090_22_Caucasian_female_neutral_frontal \| \| Rafd090_22_Caucasian_female_sad_frontal \| \| Rafd090_26_Caucasian_female_disgusted_frontal \| \| Rafd090_26_Caucasian_female_neutral_frontal \| \| Rafd090_26_Caucasian_female_sad_frontal \| \| Rafd090_31_Caucasian_female_angry_frontal \| \| Rafd090_32_Caucasian_female_angry_frontal \| \| Rafd090_32_Caucasian_female_sad_frontal \| \| Rafd090_37_Caucasian_female_neutral_frontal \| \| Rafd090_61_Caucasian_female_fearful_frontal \| \| Rafd090_61_Caucasian_female_surprised_frontal \| \| Rafd090_23_Caucasian_male_surprised_frontal \| \| Rafd090_30_Caucasian_male_angry_frontal \| \| Rafd090_36_Caucasian_male_angry_frontal \| \| Rafd090_46_Caucasian_male_disgusted_frontal \| \| Rafd090_46_Caucasian_male_fearful_frontal \| \| Rafd090_46_Caucasian_male_neutral_frontal \| \| Rafd090_47_Caucasian_male_disgusted_frontal \| \| Rafd090_47_Caucasian_male_sad_frontal \| \| Rafd090_47_Caucasian_male_surprised_frontal \| \| Rafd090_14_Caucasian_female_fearful_frontal \| \| Rafd090_15_Caucasian_male_disgusted_frontal \| \| Rafd090_18_Caucasian_female_disgusted_frontal \| \| Rafd090_19_Caucasian_female_neutral_frontal \| \| Rafd090_57_Caucasian_female_surprised_frontal \| \| Rafd090_01_Caucasian_female_neutral_frontal \| \| Rafd090_01_Caucasian_female_surprised_frontal \| \| Rafd090_02_Caucasian_female_angry_frontal \| \| Rafd090_02_Caucasian_female_surprised_frontal \| \| Rafd090_03_Caucasian_male_disgusted_frontal \| \| Rafd090_03_Caucasian_male_sad_frontal \| \| Rafd090_04_Caucasian_female_surprised_frontal \| \| Rafd090_08_Caucasian_female_surprised_frontal \| \| Rafd090_22_Caucasian_female_fearful_frontal \| \| Rafd090_22_Caucasian_female_happy_frontal \| \| Rafd090_22_Caucasian_female_surprised_frontal \| \| Rafd090_26_Caucasian_female_surprised_frontal \| \| Rafd090_27_Caucasian_female_fearful_frontal \| \| Rafd090_27_Caucasian_female_neutral_frontal \| \| Rafd090_31_Caucasian_female_neutral_frontal \| \| Rafd090_58_Caucasian_female_neutral_frontal \| \| Rafd090_61_Caucasian_female_disgusted_frontal \| \| Rafd090_23_Caucasian_male_disgusted_frontal \| \| Rafd090_25_Caucasian_male_happy_frontal \| \| Rafd090_25_Caucasian_male_surprised_frontal \| \| Rafd090_28_Caucasian_male_neutral_frontal \| \| Rafd090_30_Caucasian_male_neutral_frontal \| \| Rafd090_33_Caucasian_male_disgusted_frontal \| \| Rafd090_38_Caucasian_male_neutral_frontal \| \| Rafd090_46_Caucasian_male_angry_frontal \| \| Rafd090_71_Caucasian_male_disgusted_frontal \| \| Rafd090_71_Caucasian_male_neutral_frontal \| \| Rafd090_12_Caucasian_female_disgusted_frontal \| \| Rafd090_14_Caucasian_female_surprised_frontal \| \| Rafd090_16_Caucasian_female_sad_frontal \| \| Rafd090_16_Caucasian_female_surprised_frontal \| \| Rafd090_18_Caucasian_female_happy_frontal \| \| Rafd090_19_Caucasian_female_disgusted_frontal \| \| Rafd090_20_Caucasian_male_disgusted_frontal \| \| Rafd090_21_Caucasian_male_neutral_frontal \| \| Rafd090_01_Caucasian_female_disgusted_frontal \| \| Rafd090_01_Caucasian_female_happy_frontal \| \| Rafd090_02_Caucasian_female_disgusted_frontal \| \| Rafd090_03_Caucasian_male_happy_frontal \| \| Rafd090_03_Caucasian_male_surprised_frontal \| \| Rafd090_04_Caucasian_female_disgusted_frontal \| \| Rafd090_04_Caucasian_female_happy_frontal \| \| Rafd090_05_Caucasian_male_angry_frontal \| \| Rafd090_05_Caucasian_male_sad_frontal \| \| Rafd090_08_Caucasian_female_disgusted_frontal \| \| Rafd090_09_Caucasian_male_angry_frontal \| \| Rafd090_09_Caucasian_male_happy_frontal \| \| Rafd090_10_Caucasian_male_sad_frontal \| \| Rafd090_27_Caucasian_female_happy_frontal \| \| Rafd090_27_Caucasian_female_sad_frontal \| \| Rafd090_27_Caucasian_female_surprised_frontal \| \| Rafd090_31_Caucasian_female_disgusted_frontal \| \| Rafd090_32_Caucasian_female_happy_frontal \| \| Rafd090_32_Caucasian_female_neutral_frontal \| \| Rafd090_32_Caucasian_female_surprised_frontal \| \| Rafd090_37_Caucasian_female_sad_frontal \| \| Rafd090_56_Caucasian_female_disgusted_frontal \| \| Rafd090_58_Caucasian_female_surprised_frontal \| \| Rafd090_23_Caucasian_male_angry_frontal \| \| Rafd090_23_Caucasian_male_sad_frontal \| \| Rafd090_24_Caucasian_male_neutral_frontal \| \| Rafd090_25_Caucasian_male_sad_frontal \| \| Rafd090_28_Caucasian_male_fearful_frontal \| \| Rafd090_28_Caucasian_male_happy_frontal \| \| Rafd090_30_Caucasian_male_surprised_frontal \| \| Rafd090_33_Caucasian_male_neutral_frontal \| \| Rafd090_33_Caucasian_male_sad_frontal \| \| Rafd090_33_Caucasian_male_surprised_frontal \| \| Rafd090_36_Caucasian_male_happy_frontal \| \| Rafd090_36_Caucasian_male_surprised_frontal \| \| Rafd090_38_Caucasian_male_happy_frontal \| \| Rafd090_38_Caucasian_male_surprised_frontal \| \| Rafd090_46_Caucasian_male_sad_frontal \| \| Rafd090_49_Caucasian_male_disgusted_frontal \| \| Rafd090_49_Caucasian_male_neutral_frontal \| \| Rafd090_49_Caucasian_male_surprised_frontal \| \| Rafd090_71_Caucasian_male_fearful_frontal \| \| Rafd090_71_Caucasian_male_sad_frontal \| \| Rafd090_71_Caucasian_male_surprised_frontal \| \| Rafd090_15_Caucasian_male_neutral_frontal \| \| Rafd090_16_Caucasian_female_happy_frontal \| \| Rafd090_18_Caucasian_female_sad_frontal \| \| Rafd090_20_Caucasian_male_sad_frontal \| \| Rafd090_57_Caucasian_female_disgusted_frontal \| \| Rafd090_01_Caucasian_female_angry_frontal \| \| Rafd090_01_Caucasian_female_sad_frontal \| \| Rafd090_02_Caucasian_female_happy_frontal \| \| Rafd090_04_Caucasian_female_sad_frontal \| \| Rafd090_05_Caucasian_male_happy_frontal \| \| Rafd090_05_Caucasian_male_neutral_frontal \| \| Rafd090_07_Caucasian_male_happy_frontal \| \| Rafd090_07_Caucasian_male_neutral_frontal \| \| Rafd090_08_Caucasian_female_happy_frontal \| \| Rafd090_08_Caucasian_female_neutral_frontal \| \| Rafd090_10_Caucasian_male_happy_frontal \| \| Rafd090_10_Caucasian_male_surprised_frontal \| \| Rafd090_22_Caucasian_female_disgusted_frontal \| \| Rafd090_26_Caucasian_female_happy_frontal \| \| Rafd090_31_Caucasian_female_happy_frontal \| \| Rafd090_31_Caucasian_female_sad_frontal \| \| Rafd090_31_Caucasian_female_surprised_frontal \| \| Rafd090_37_Caucasian_female_angry_frontal \| \| Rafd090_37_Caucasian_female_happy_frontal \| \| Rafd090_56_Caucasian_female_happy_frontal \| \| Rafd090_56_Caucasian_female_sad_frontal \| \| Rafd090_58_Caucasian_female_angry_frontal \| \| Rafd090_58_Caucasian_female_happy_frontal \| \| Rafd090_58_Caucasian_female_sad_frontal \| \| Rafd090_61_Caucasian_female_happy_frontal \| \| Rafd090_61_Caucasian_female_sad_frontal \| \| Rafd090_23_Caucasian_male_happy_frontal \| \| Rafd090_23_Caucasian_male_neutral_frontal \| \| Rafd090_24_Caucasian_male_happy_frontal \| \| Rafd090_28_Caucasian_male_sad_frontal \| \| Rafd090_30_Caucasian_male_happy_frontal \| \| Rafd090_33_Caucasian_male_happy_frontal \| \| Rafd090_36_Caucasian_male_sad_frontal \| \| Rafd090_38_Caucasian_male_disgusted_frontal \| \| Rafd090_38_Caucasian_male_sad_frontal \| \| Rafd090_46_Caucasian_male_happy_frontal \| \| Rafd090_47_Caucasian_male_happy_frontal \| \| Rafd090_49_Caucasian_male_happy_frontal \| \| Rafd090_49_Caucasian_male_sad_frontal \| \| Rafd090_71_Caucasian_male_happy_frontal \| \| Rafd090_12_Caucasian_female_happy_frontal \| \| Rafd090_12_Caucasian_female_neutral_frontal \| \| Rafd090_12_Caucasian_female_surprised_frontal \| \| Rafd090_14_Caucasian_female_happy_frontal \| \| Rafd090_15_Caucasian_male_happy_frontal \| \| Rafd090_15_Caucasian_male_surprised_frontal \| \| Rafd090_19_Caucasian_female_happy_frontal \| \| Rafd090_20_Caucasian_male_happy_frontal \| \| Rafd090_20_Caucasian_male_surprised_frontal \| \| Rafd090_21_Caucasian_male_happy_frontal \| \| Rafd090_57_Caucasian_female_happy_frontal \| \| Rafd090_57_Caucasian_female_sad_frontal \| | \| 5 \| \| --- \| \| 25 \| \| 35 \| \| 35 \| \| 40 \| \| 40 \| \| 40 \| \| 40 \| \| 45 \| \| 45 \| \| 45 \| \| 50 \| \| 50 \| \| 55 \| \| 55 \| \| 55 \| \| 55 \| \| 60 \| \| 60 \| \| 60 \| \| 60 \| \| 60 \| \| 60 \| \| 60 \| \| 60 \| \| 60 \| \| 60 \| \| 65 \| \| 65 \| \| 65 \| \| 65 \| \| 65 \| \| 65 \| \| 65 \| \| 65 \| \| 65 \| \| 65 \| \| 65 \| \| 65 \| \| 70 \| \| 70 \| \| 70 \| \| 70 \| \| 70 \| \| 70 \| \| 70 \| \| 70 \| \| 70 \| \| 70 \| \| 75 \| \| 75 \| \| 75 \| \| 75 \| \| 75 \| \| 75 \| \| 75 \| \| 75 \| \| 75 \| \| 75 \| \| 75 \| \| 75 \| \| 75 \| \| 75 \| \| 75 \| \| 75 \| \| 75 \| \| 75 \| \| 75 \| \| 75 \| \| 75 \| \| 80 \| \| 80 \| \| 80 \| \| 80 \| \| 80 \| \| 80 \| \| 80 \| \| 80 \| \| 80 \| \| 80 \| \| 80 \| \| 80 \| \| 80 \| \| 80 \| \| 80 \| \| 80 \| \| 80 \| \| 80 \| \| 80 \| \| 80 \| \| 80 \| \| 80 \| \| 80 \| \| 80 \| \| 80 \| \| 80 \| \| 80 \| \| 80 \| \| 80 \| \| 80 \| \| 80 \| \| 80 \| \| 80 \| \| 80 \| \| 85 \| \| 85 \| \| 85 \| \| 85 \| \| 85 \| \| 85 \| \| 85 \| \| 85 \| \| 85 \| \| 85 \| \| 85 \| \| 85 \| \| 85 \| \| 85 \| \| 85 \| \| 85 \| \| 85 \| \| 85 \| \| 85 \| \| 85 \| \| 85 \| \| 85 \| \| 85 \| \| 85 \| \| 85 \| \| 85 \| \| 85 \| \| 85 \| \| 85 \| \| 85 \| \| 85 \| \| 85 \| \| 85 \| \| 90 \| \| 90 \| \| 90 \| \| 90 \| \| 90 \| \| 90 \| \| 90 \| \| 90 \| \| 90 \| \| 90 \| \| 90 \| \| 90 \| \| 90 \| \| 90 \| \| 90 \| \| 90 \| \| 90 \| \| 90 \| \| 90 \| \| 90 \| \| 90 \| \| 90 \| \| 90 \| \| 90 \| \| 90 \| \| 90 \| \| 90 \| \| 90 \| \| 90 \| \| 90 \| \| 90 \| \| 90 \| \| 90 \| \| 90 \| \| 90 \| \| 95 \| \| 95 \| \| 95 \| \| 95 \| \| 95 \| \| 95 \| \| 95 \| \| 95 \| \| 95 \| \| 95 \| \| 95 \| \| 95 \| \| 95 \| \| 95 \| \| 95 \| \| 95 \| \| 95 \| \| 95 \| \| 95 \| \| 95 \| \| 95 \| \| 95 \| \| 95 \| \| 95 \| \| 95 \| \| 95 \| \| 95 \| \| 95 \| \| 95 \| \| 95 \| \| 95 \| \| 95 \| \| 95 \| \| 95 \| \| 95 \| \| 95 \| \| 95 \| \| 95 \| \| 95 \| \| 95 \| \| 95 \| \| 95 \| \| 95 \| \| 95 \| \| 95 \| \| 95 \| \| 95 \| \| 95 \| \| 95 \| \| 100 \| \| 100 \| \| 100 \| \| 100 \| \| 100 \| \| 100 \| \| 100 \| \| 100 \| \| 100 \| \| 100 \| \| 100 \| \| 100 \| \| 100 \| \| 100 \| \| 100 \| \| 100 \| \| 100 \| \| 100 \| \| 100 \| \| 100 \| \| 100 \| \| 100 \| \| 100 \| \| 100 \| \| 100 \| \| 100 \| \| 100 \| \| 100 \| \| 100 \| \| 100 \| \| 100 \| \| 100 \| \| 100 \| \| 100 \| \| 100 \| \| 100 \| \| 100 \| \| 100 \| \| 100 \| \| 100 \| \| 100 \| \| 100 \| \| 100 \| \| 100 \| \| 100 \| \| 100 \| \| 100 \| \| 100 \| \| 100 \| \| 100 \| \| 100 \| \| 100 \| | \| 36.364 \| \| --- \| \| 63.636 \| \| 58.333 \| \| 87.5 \| \| 60.87 \| \| 83.333 \| \| 68.182 \| \| 30.435 \| \| 40.909 \| \| 60.87 \| \| 83.333 \| \| 38.095 \| \| 91.667 \| \| 72.727 \| \| 95.833 \| \| 92 \| \| 62.963 \| \| 83.333 \| \| 100 \| \| 95.833 \| \| 91.667 \| \| 77.273 \| \| 54.167 \| \| 72 \| \| 96 \| \| 76.19 \| \| 75 \| \| 81.818 \| \| 77.273 \| \| 86.957 \| \| 79.167 \| \| 68 \| \| 91.667 \| \| 100 \| \| 92.308 \| \| 86.957 \| \| 68.182 \| \| 88 \| \| 95.833 \| \| 90.909 \| \| 83.333 \| \| 80 \| \| 91.304 \| \| 95.652 \| \| 84 \| \| 77.273 \| \| 95.455 \| \| 85.714 \| \| 83.333 \| \| 75 \| \| 73.913 \| \| 95.652 \| \| 90.909 \| \| 75 \| \| 70.833 \| \| 85.714 \| \| 91.304 \| \| 71.429 \| \| 83.333 \| \| 92 \| \| 91.667 \| \| 88.462 \| \| 80.769 \| \| 79.167 \| \| 95 \| \| 100 \| \| 86.957 \| \| 95.652 \| \| 52 \| \| 95.833 \| \| 100 \| \| 91.304 \| \| 91.667 \| \| 100 \| \| 96 \| \| 60.87 \| \| 86.364 \| \| 91.304 \| \| 66.667 \| \| 86.957 \| \| 91.304 \| \| 96 \| \| 95.652 \| \| 79.167 \| \| 95.833 \| \| 100 \| \| 65.385 \| \| 100 \| \| 77.273 \| \| 95.455 \| \| 86.364 \| \| 82.609 \| \| 70.37 \| \| 92.308 \| \| 69.231 \| \| 86.364 \| \| 90.909 \| \| 86.364 \| \| 86.364 \| \| 100 \| \| 61.538 \| \| 92.308 \| \| 75 \| \| 75 \| \| 91.304 \| \| 91.667 \| \| 82.609 \| \| 86.957 \| \| 83.333 \| \| 76 \| \| 86.957 \| \| 82.609 \| \| 100 \| \| 100 \| \| 75 \| \| 100 \| \| 78.261 \| \| 91.304 \| \| 88 \| \| 80 \| \| 100 \| \| 100 \| \| 77.273 \| \| 95.652 \| \| 91.304 \| \| 87.5 \| \| 81.818 \| \| 90.909 \| \| 83.333 \| \| 83.333 \| \| 91.304 \| \| 100 \| \| 92 \| \| 68.182 \| \| 82.609 \| \| 88.462 \| \| 75 \| \| 91.304 \| \| 86.364 \| \| 95.833 \| \| 87.5 \| \| 82.609 \| \| 86.957 \| \| 95.833 \| \| 96 \| \| 95.833 \| \| 100 \| \| 92 \| \| 100 \| \| 91.304 \| \| 100 \| \| 95.652 \| \| 86.364 \| \| 68.182 \| \| 78.261 \| \| 100 \| \| 95.833 \| \| 88 \| \| 91.667 \| \| 87.5 \| \| 85.714 \| \| 95.652 \| \| 95 \| \| 100 \| \| 81.818 \| \| 92 \| \| 100 \| \| 95.833 \| \| 95.652 \| \| 69.231 \| \| 68.182 \| \| 80.769 \| \| 75 \| \| 95.455 \| \| 86.957 \| \| 100 \| \| 91.304 \| \| 78.261 \| \| 100 \| \| 100 \| \| 43.478 \| \| 84 \| \| 95.652 \| \| 100 \| \| 95.833 \| \| 100 \| \| 95.652 \| \| 95.652 \| \| 78.261 \| \| 96.154 \| \| 100 \| \| 100 \| \| 100 \| \| 95.652 \| \| 100 \| \| 100 \| \| 95.455 \| \| 96 \| \| 78.261 \| \| 92 \| \| 100 \| \| 100 \| \| 100 \| \| 92.308 \| \| 95.833 \| \| 100 \| \| 91.667 \| \| 100 \| \| 86.364 \| \| 100 \| \| 73.077 \| \| 92.308 \| \| 88.889 \| \| 85 \| \| 100 \| \| 90.476 \| \| 100 \| \| 100 \| \| 95.652 \| \| 95.652 \| \| 79.167 \| \| 100 \| \| 96 \| \| 100 \| \| 95.833 \| \| 91.304 \| \| 95.455 \| \| 100 \| \| 100 \| \| 96 \| \| 100 \| \| 100 \| \| 95.455 \| \| 86.364 \| \| 100 \| \| 100 \| \| 100 \| \| 95.652 \| \| 100 \| \| 100 \| \| 100 \| \| 100 \| \| 100 \| \| 100 \| \| 95.455 \| \| 100 \| \| 90.909 \| \| 100 \| \| 100 \| \| 96 \| \| 92 \| \| 100 \| \| 100 \| \| 80 \| \| 86.364 \| \| 90.909 \| \| 95.833 \| \| 100 \| \| 100 \| \| 84.615 \| \| 100 \| \| 100 \| \| 100 \| \| 100 \| \| 100 \| \| 100 \| \| 100 \| \| 100 \| \| 95.455 \| \| 86.957 \| \| 96 \| \| 100 \| \| 95.833 \| |
